# Goal-directed upper limb movement patterns and hand grip forces in multiple sclerosis

**DOI:** 10.1177/20552173221116272

**Published:** 2022-08-11

**Authors:** Christoph M Kanzler, Ramona Sylvester, Roger Gassert, Jan Kool, Olivier Lambercy, Roman Gonzenbach

**Affiliations:** Rehabilitation Engineering Laboratory, Institute of Robotics and Intelligent Systems, Department of Health Sciences and Technology, 27219 ETH Zurich, Zurich, Switzerland; Future Health Technologies, Singapore-ETH Centre, Campus for Research Excellence And Technological Enterprise (CREATE), Singapore; Rehabilitation Center Valens, Valens, Switzerland; Rehabilitation Engineering Laboratory, Institute of Robotics and Intelligent Systems, Department of Health Sciences and Technology, 27219 ETH Zurich, Zurich, Switzerland; Future Health Technologies, Singapore-ETH Centre, Campus for Research Excellence And Technological Enterprise (CREATE), Singapore; Rehabilitation Center Valens, Valens, Switzerland; Rehabilitation Engineering Laboratory, Institute of Robotics and Intelligent Systems, Department of Health Sciences and Technology, 27219 ETH Zurich, Zurich, Switzerland; Future Health Technologies, Singapore-ETH Centre, Campus for Research Excellence And Technological Enterprise (CREATE), Singapore; Rehabilitation Center Valens, Valens, Switzerland

**Keywords:** digital health metrics, upper limb, motor control, multiple sclerosis, movement pattern, grip forces

## Abstract

**Background:**

Upper limb disability in persons with Multiple Sclerosis (pwMS) leads to increased dependence on caregivers. To better understand upper limb disability, observer-based or time-based clinical assessments have been applied. However, these only poorly capture the behavioural aspects underlying goal-directed task performance.

**Objective:**

We aimed to document alterations in goal-directed upper limb movement patterns and hand grip forces in a cohort of pwMS (n = 123) with mild to moderate upper limb impairments.

**Methods:**

We relied on the Virtual Peg Insertion Test (VPIT), a technology-aided assessment with a goal-directed pick-and-place task providing a set of validated digital health metrics.

**Results:**

All metrics indicated significant differences to an able-bodied reference sample (p < 0.001), with smoothness, speed, and grip force control during object manipulation being most affected in pwMS. Such abnormalities negatively influenced the time to complete the goal-directed task (p < 0.001, R^2^ = 0.77), thereby showing their functional relevance. Lastly, abnormalities in movement patterns and grip force control were consistently found even in pwMS with clinically normal gross dexterity and grip strength.

**Conclusion:**

This work provides a systematic documentation on goal-directed upper limb movement patterns and hand grip forces in pwMS, ultimately paving the way for an early detection of MS sign using digital health metrics.

## Introduction

Persons with multiple sclerosis (pwMS) commonly experience sensorimotor impairments in arm and hand that prohibit smooth and efficient goal-directed tasks.^1–3^ These impairments are a key factor contributing to reduced independence and quality of life in pwMS, thus emphasizing their importance to patients, researchers, and healthcare providers.^4^

Sensorimotor impairments in pwMS have been documented using observer- or rater-based clinical assessments, allowing to identify, for example, weakness, abnormal muscle tone, fatigue, and tremor as common symptoms.^1^ In addition, clinical assessments were applied to determine the ability to perform goal-directed tasks, such as the Nine Hole Peg Test (NHPT) or Box and Block Test (BBT).^5^ Based on the time to perform such tasks, abnormalities in prehension, fine and gross dexterity, and coordination have been identified in pwMS.^2^

While these studies provide first insights into upper limb impairments and activity capacity, they are limited by the applied assessment methodologies.^6^ Specifically, clinical assessments of activities are not able to delineate between different impairments that can lead to abnormal behavior in the task. For example, a person can have abnormal performance in the BBT or NHPT, but it is unclear whether this is resulting from, for example, suboptimal movement efficiency or impaired grip force control. Moreover, conventional clinical assessments are not able to quantify movement quality, which is one of the behavioural hallmark features of goal-directed movements.^7^

Technology-based assessments with instrumented goal-directed tasks can record objective data on upper limb movement patterns and hand grip forces.^8^ These can be processed into digital health metrics objectively quantifying movement quality, including speed, efficiency, and smoothness, as well as grip force control.^9^ This promises novel insights into the mechanisms of upper limb sensorimotor impairments and their relation to activity capacity. While technology-based assessments already helped to advance the understanding of gait abnormalities in pwMS,^10^ their application to complex upper limb movements and related impairments is still in its infancy, as mainly small pilot studies were conducted to show the feasibility of such assessments.^11,12^

The aim of this work is to document alterations in goal-directed upper limb movement patterns and hand grip forces in a cohort of pwMS with mild to moderate upper limb impairments. For this purpose, we relied on the Virtual Peg Insertion Test (VPIT), a technology-based assessment featuring a goal-directed pick-and-place task that provides validated digital health metrics describing movement patterns and hand grip forces.^9^

We hypothesized that pwMS consistently show various level of abnormal movement patterns and hand grip forces and that these negatively influence goal-directed task performance. Also, we expected to identify abnormal movement patterns and hand grip forces in pwMS that do not show abnormalities in standard clinical scales thanks to the high sensitivity of the digital health metrics. This would help to advance our understanding of abnormal upper limb behavior in pwMS and could provide first evidence for the clinical benefit of digital health metrics.

## Methods

### Participants and procedures

Adults with a confirmed diagnosis of MS according to the McDonald criteria that were admitted to the in-patient program of the Rehabilitation Center Valens (Valens, Switzerland) were considered for this study.^13^ Exclusion criteria were the presence of severe disability (Expanded Disability Status Scale (EDSS) > 7.5), inability to follow procedures, and concomitant diseases potentially affecting upper limb function. All procedures were approved by the local Ethics Committee (EKOS 2020–02212) and implemented in accordance with ethical standards and the Declaration of Helsinki.

### Virtual Peg Insertion Test (VPIT)

The VPIT as a platform to study upper limb movement patterns and hand grip force control has been described in-depth in previous work.^9,14,15^ In brief, the VPIT consists of a robotic end-effector (Touch, 3D Systems, US), a force sensing handle, and a laptop displaying a virtual pick-and-place task. The task requires the insertion of nine virtual pegs in nine virtual holes by coordinating movements and hand grip forces (details in supplementary materials (SM)). The VPIT protocol consists of an initial familiarization period with standardized instructions followed by five repetitions of the task (i.e. insertion of all pegs five times with breaks in-between). Herein, we applied a shortened version of the protocol with only three repetitions of the task that has recently been validated and shown to provide a good trade-off between robustness and clinical applicability.^16^ The VPIT was applied to the left and right upper extremity given that pwMS can exhibit abnormalities on both body sides. Participants were instructed to complete this the VPIT as fast and accurately as possible.

In order to extract digital health metrics from the kinematic and kinetic data, a signal processing framework was previously developed and validated.^9,15,16^ In brief, the sensor data is first pre-processed and temporally segmented into different task phases that are supposed to capture unique behavioural aspects of the tasks. This includes the *transport* (gross movement between peg pickup and peg insertion), *return* (gross movement between peg insertion and next peg pickup), *peg approach* (fine movement before lifting a peg), and *hole approach* (fine movement before inserting a peg) phases.

Subsequently, sensor-based core metrics are extracted from these phases to describe movement smoothness, speed, accuracy, efficiency, and grip force control. For the applied protocol with three task repetitions, a core set of eight digital health metrics has been previously identified and validated,^16^ based on their pathophysiological motivation and clinimetric properties (test-retest reliability, measurement error, learning effects, inter-correlations, discriminative power).^9^ The core metrics (details in SM) include the normalized logarithmic jerk metric (*log jerk transport/return)* and the spectral arc length metric (*SPARC return*) as measures of movement smoothness,^7,9^ the ratio between the shortest distance between start and target and the actually covered distance (*path length ratio transport*) to describe movement efficiency,^8,9^ and the maximum velocity metric (*velocity max. return*) as a descriptor of movement speed*.* Further, three metrics were calculated based on the change in grip force (*force rate)* to describe the smoothness of grip force control (*grip force rate num. peaks transport, grip force rate SPARC transport, and grip force rate SPARC hole approach)*. The metrics are expressed as *normalized VPIT scores* (0% median of reference sample, 100% worst participant in initial VPIT database, negative values better performance than reference sample, details in SM). This normalization step also aims to remove potential effects of demographics, such as age and sex, to allow population-level comparison of the reference sample and pwMS.^9^

### Conventional clinical assessments

Conventional assessments were performed to provide a clinical characterization of disability. Specifically, the EDSS was used to describe the overall level of disability (0: normal neurological exam; 10: death due to MS).^17^ Further, upper limb gross dexterity was described using the Box and Block Test (BBT).^18^ Lastly, upper limb grip strength was tested with the Jamar dynamometer.^19^

### Data analysis

To allow an intuitive visual inspection of the raw data collected with the VPIT, velocity traces and grip force rates were visualized on a group level for the *transport* and *return* phase of the task (details in SM) and statistical differences were analyzed using statistical parametric mapping.^20^ Next, abnormal behavior in the VPIT was analyzed in-depth using the defined core set of digital health metrics. On a group level, all metrics were statistically compared between pwMS and the normative sample using mixed effect models (details in SM).

Further, individual pwMS showing abnormalities in a digital health metric were identified by using the 95^th^-percentile threshold of the normative sample, which was introduced in previous work.^9^ A subgroup analysis was done in pwMS that have normal gross dexterity and grip strength, as defined by the BBT and grip strength measurements (i.e. pwMS are within the 95^th^-percentile of publicly available normative sample). Lastly, the influence of abnormal upper limb movement patterns and hand grip forces on the VPIT task completion time were evaluated using a mixed effect model (details in SM).

## Results

For the duration of the study, 318 pwMS were admitted to the Rehabilitation Center Valens of which 147 were included in the study. However, eleven of those did not consent with the further usage of their data for research purposes and were excluded for analysis. While all remaining pwMS attempted to complete the VPIT protocol with both body sides, 13 pwMS had insufficient upper limb function or too severe cognitive deficits to complete the protocol with at least one body side. Thus, VPIT and clinical data was collected in total from 123 pwMS (53.0 ± 14.5 years, BBT 55 ± 16 block/min, grip strength 24.7 ± 9.6 kg, details in Table 1). This led to a total of 233 observations (i.e. either right or left body side of a pwMS) that were used for further analysis.

**Table 1.** Population description.

Characteristics	Value
Number of pwMS	123
Number of available data points (including left and right body side)	233
Age	53.0 ± 14.5 (19–76)
Sex	40 male, 83 female
MS type (2 missing values)	33 primary progressive 34 secondary progress 54 relapsing-remitting
Time since diagnosis (years)	13.4–14.1 (0.3–43.4)
Expanded Disability Status Scale (0–10) (6 missing values)	5.0 ± 2.0 (2.0–7.5)
Box and Block Test (blocks per minute) (2 missing values)	55 ± 16 (16–83)
Grip strength (kg)	24.7 ± 9.6 (0–70.7)

Values denoted as median ± interquartile range. MS: multiple sclerosis; pwMS: persons with multiple sclerosis

**Figure 1.**
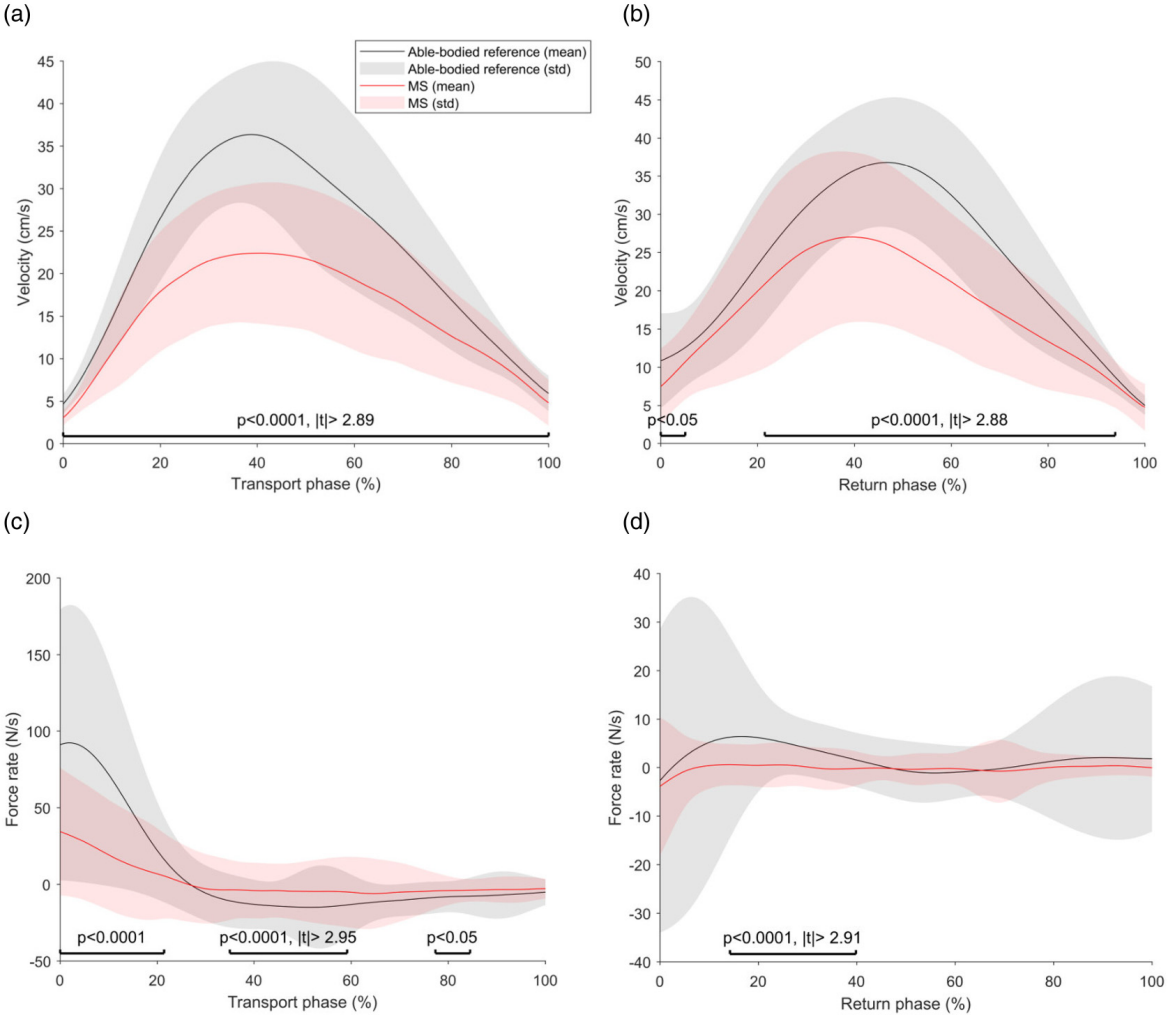
**Group-level velocity and grip force rate data.** PwMS show reduced movement speed and reduced ability to dynamically adapt grip forces during a goal-directed task. (a): Velocity transport. (b): Velocity return. (c): Grip force rate transport. (d): Grip force rate return.

**Figure 2.**
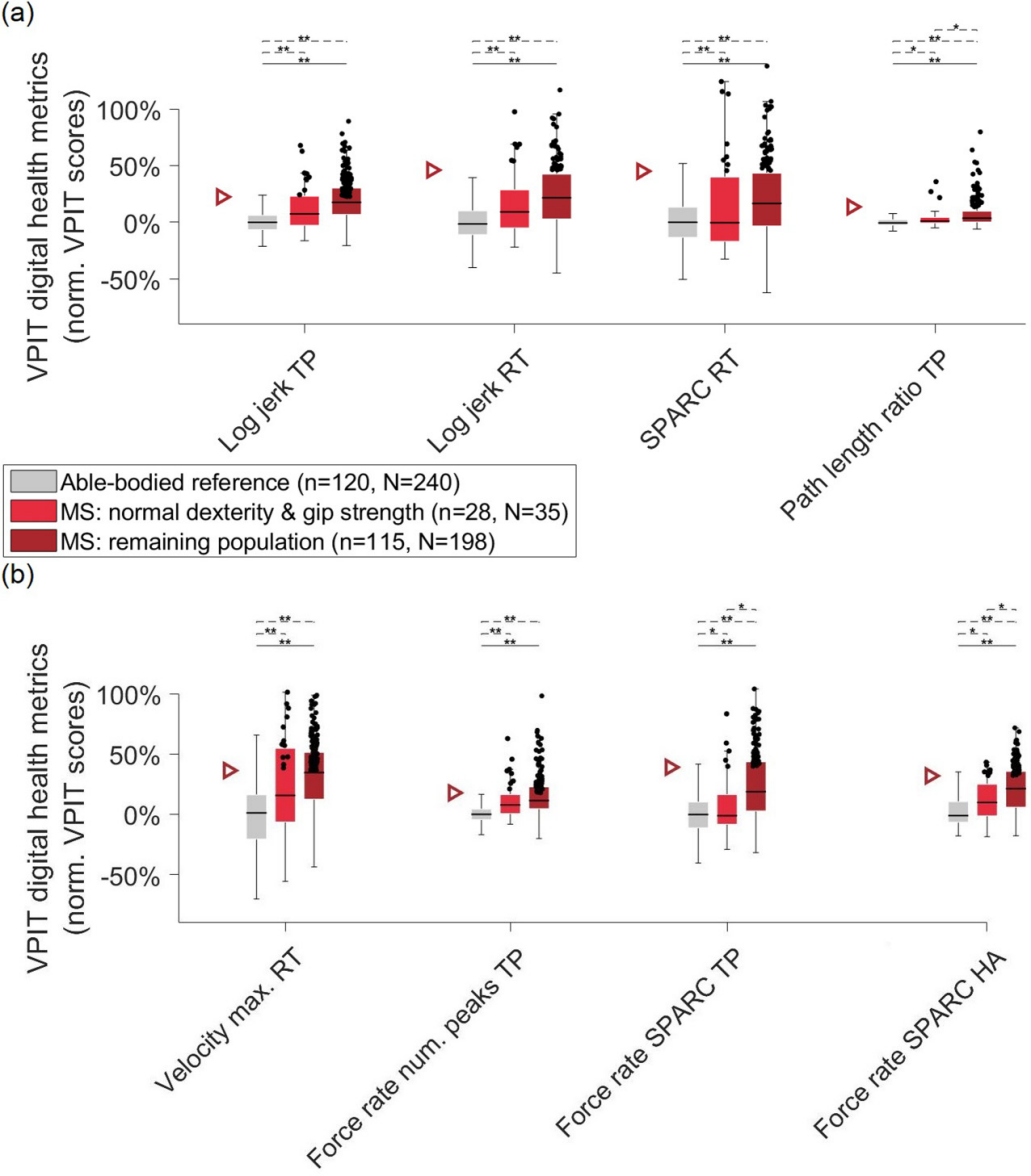
**Group-level analysis of digital health metrics from the VPIT.** An age- and sex-corrected able-bodied reference sample, a MS sample that has normal gross dexterity (Box and Block Test) and grip strength, and the remaining MS sample were visualized. 0% indicates the median of an able-bodied reference sample. 100% indicates the worst neurological participant in the initial VPIT database. Negative values indicate performance better than the median of the reference sample. The triangle indicates the abnormal behaviour threshold (i.e. 95^th^-percentile of able-bodied reference) and all observations above the threshold are visualized as black dots. TP: Transport. RT: Return. HA: Hole approach. SPARC: Spectral arc length. *p < 0.05, **p < 0.001. n refers to the number of participants, N refers to the number of observations.

The group-level velocity and grip force rate data are visualized in Figure 1, showing significant differences between able-bodied participants and pwMS for movement speed and grip force control during all task phases. When analyzing the digital health metrics on a group level, the pwMS performed significantly worse in all aspects of upper limb movement patterns and grip forces (Figure 2). The percentage of observations that showed abnormalities (i.e. worse than 95^th^-percentile) in metrics describing movement smoothness was 37.8% for *log jerk transport*, 19.6% for *log jerk return*, and 23.9% for *SPARC return*. For the *path length ratio* *transport* metric describing movement efficiency, 16.1% of observations were identified as abnormal*,* whereas for the *velocity max. return* metric describing movement speed 46.1% of observations were abnormal*. Lastly*, for metrics describing grip force control, 33.0% of observations were abnormal for *grip force rate num. peaks transport,* 25.7% *for grip force rate SPARC,* and 30.9% for *grip force rate SPARC hole approach*. Out of all observations, 75.7% were impaired in at least one digital health metric, 58.7% in at least two metrics, 40.0% in at least three metrics, 27.0% in at least four metrics, 16.1% in at least five metrics, 10.4% in at least six metrics, 4.8% in at least seven metrics, and 0.4% in eight metrics.

**Figure 3.**
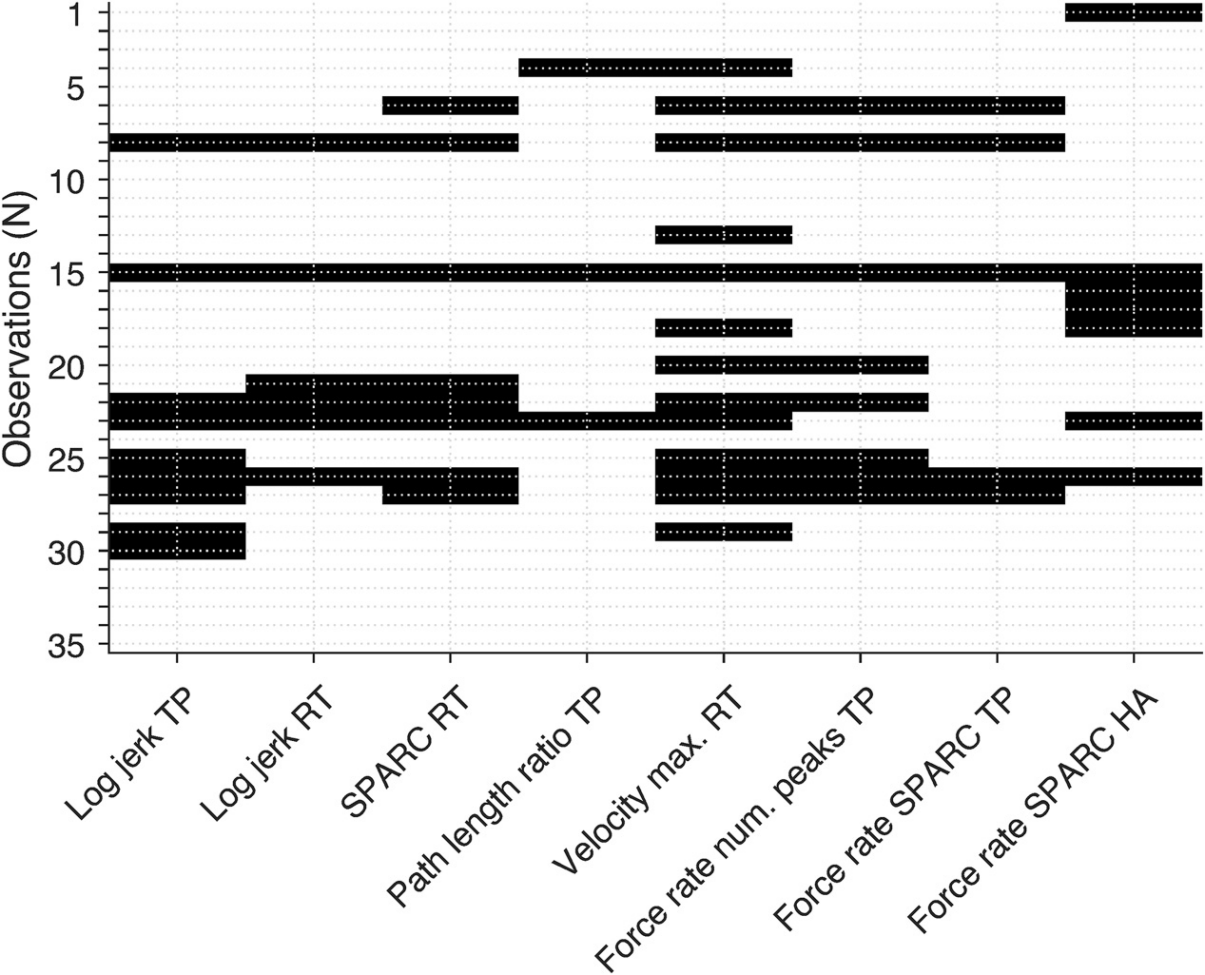
**Abnormalities (black) according to the digital health metrics in pwMS with clinically normal gross dexterity and grip strength.** Abnormalities are present if the value of a metric is outside the 95^th^-percentile of an able-bodied reference sample. Observations refers to either the left or right body side of pwMS. TP: Transport. RT: Return. HA: Hole approach. SPARC: Spectral arc length.

Figure 3 displays the digital health metrics that indicate abnormalities in pwMS, which are within the norm of the BBT and the grip strength measurements (28 patients, 35 observations). Within this sample, the percentage of observations showing abnormalities for metrics describing movement smoothness was 25.7% for *log jerk transport*, 17.1% for *log jerk return*, 22.9% for *SPARC return.* For the *path length ratio transport* metric describing movement efficiency, 8.6% of observations were abnormal, whereas *37.1%* were abnormal for the metric *velocity max. return* describing movement speed. Lastly, for metrics describing grip force control, 22.9% of observations were abnormal for *grip force rate num. peaks transport,* 14.3% *for grip force rate SPARC,* and 20.0% for *grip force rate SPARC hole approach*. 51.4% of observations in this sample were impaired in at least one digital health metric, 37.1% in at least two metrics, 22.9% in at least three metrics, 20.0% in at least four metrics, 17.1% in at least five metrics, 11.4% in at least six metrics, 5.7% in at least seven metrics, and 2.9% in eight metrics.

When analyzing how abnormal movement patterns and hand grip forces influence goal-directed task performance, the mixed effect model analysis (adjusted-R^2^ value 0.77, details in Table SM1 and Figure SM1) showed that the metrics significantly influencing the VPIT task completion time were *log jerk transport* (*t* = 2.07, p < 0.05), *velocity max. return* (*t* = 3.7, p < 0.001), the *grip force rate SPARC hole approach* (*t* *=* 7.2, p < 0.001), and the *path length ratio transport* (t = 6.3, p < 0.001).

## Discussion

So far, a comprehensive analysis of goal-directed upper limb kinematics and kinetics is lacking in pwMS. Herein, we aimed to fill this gap by applying an end effector-based robotic assessment providing validated digital health metrics^9^ to 123 pwMS. Our main findings were that pwMS frequently exhibit abnormal movement patterns and hand grip forces, with smoothness, speed, and force control during object insertion being most commonly impaired. Additionally, we observed that such abnormalities explain large variability in the time to perform a goal-directed task, thereby underlining their functional relevance. Lastly, we found that a majority of pwMS with clinically normal gross dexterity and grip strength consistently still exhibit abnormalities in movement patterns and hand grip forces.

This work provides a systematic documentation of abnormalities in upper limb movement patterns and hand grip forces in a cohort of pwMS with mild to moderate upper limb disability undergoing in-patient rehabilitation in Switzerland. This was highly warranted, after multiple studies separately focusing on upper limb movements or grip forces in small groups of pwMS.^21–25^

### Abnormal goal-directed upper limb movement patterns and hand grip forces in pwMS

All analyzed digital health metrics showed significantly worse performance of pwMS than a reference sample, most strongly and frequently in smoothness, speed, and force control during fine manipulations. Abnormal smoothness during goal-directed movements is reflected by the presence of sub-movements in the velocity profile.^7^ From a physiological perspective, it likely stems from abnormal feed-forward control and the following need for sensory feedback-driven adjustments towards the second half of the goal-directed movement creating multiple sub-movements.^7^ This is supported by a strong deviation of the velocity profile of pwMS from the reference sample, especially in the second half of the transport and return phase of the goal-directed task (Figure 2). However, group-level velocity profiles (Figure 2) did not reveal multiple submovements in pwMS, which is likely an artifact of the interpolation and averaging operations needed for this visualization only. Also, movement speed was more strongly affected in pwMS than movement efficiency. Given that these two constructs are linked through the speed-accuracy trade-off, this suggests that pwMS prioritize to maintain accuracy at the cost of speed in this task. The reduction of speed can be interpreted as a compensatory strategy of pwMS to cope with their reduced cognitive information processing capabilities,^26^ thus allowing them to focus on the task goals (i.e. successfully inserting all virtual pegs). This reduction in speed likely leads to higher reliance on sensory feedback loops, thereby also contributing to the observed increased number of sub-movements. An alternative interpretation would be that impairments in sensory processing and integration, for example of proprioceptive information, directly lead to reduced movement speed. Even though sensory impairments are reported in up to 85% of pwMS already in the first year of the disease,^27^ proprioceptive impairments can often be partially or fully compensated by vision,^28^ thereby making this explanation less likely. Grip strength and movement speed were only very weakly correlated (details in SM), suggesting that weakness is only a minor contributor to reduced movement speed. Force control was less dynamic and less smooth during all task phases, most strongly during peg insertion. This could be a result of sensory impairments as, during peg insertion, these abnormalities are exaggeratedas task complexity increases and cognitive-sensory loops come into play.^29^ Interestingly, abnormalities in movement smoothness during peg transport, movement efficiency during peg transport, and movement speed during the return phase as well as grip force control during fine manipulations were significantly increasing the time to complete the technology-aided assessment task. While an influence of movement efficiency and speed on the task completion time is intuitive, our results indicate that smoothness is of functional relevance in this sample of pwMS and optimizing movement smoothness during neurorehabilitation might therefore be an interesting opportunity to improve functional capabilities of pwMS. The significant influence of grip force control during fine manipulations on the task completion time further underlines the importance of this aspect of sensorimotor control in pwMS.

These results complement previous insights, which detected increased grip force magnitudes and abnormal force-load coupling in pwMS,^24,25^ and help to better connect impaired aspects of sensorimotor control and their functional relevance.

Notably, a recent study relied on an upper limb exoskeleton with a battery of weight-supported movement and limb matching tasks to characterize sensorimotor and cognitive impairments in 46 pwMS.^11^ The study found frequent impairments in pwMS in robotic motor and cognitive tasks. This is in general in line with our results, even though exact comparisons are challenged by Simmatis et al. relying on a task-based analysis whereas we focus on a metric-based analysis. Also, our study relies on an end-effector-based approach to study arm and hand impairments, thereby complementing the exoskeleton-based approach focusing on arm impairments. Recently, tablet- and smartphone-based approaches that promise frequent, unsupervised, and remote assessments of hand and finger movements have been validated in pwMS.^30–32^ In the future, it needs to be evaluated how such approaches compare to highly standardized assessments based on robotic devices that also include proximal movements and can record hand grip forces.

### Subtle alterations in pwMS with normal gross dexterity and grip strength

Intriguingly, abnormal movement speed, movement efficiency, and grip force control were frequently impaired even in pwMS with normal gross dexterity and grip strength, as measured by two clinically accepted assessments. Across most metrics, clear trends could be observed on a group-level, with pwMS with normal gross dexterity and grip strength having worse task performance than able-bodied participants and better performance than the remaining sample of pwMS. This can be explained by the sensitivity of the digital health metrics being able to capture subtle abnormalities not reflected in the clinical assessments. In addition, certain pwMS with normal gross dexterity and grip strength reached similar abnormal task performance levels than the overall worst performing pwMS (e.g. for movement speed and smoothness). This suggests that certain pwMS achieve normal performance in the BBT by relying on compensatory movement strategies, which are revealed by the digital health metrics. Also, the presence of abnormal grip force control while having normal grip strength adds on the accumulating evidence that these two aspects of upper limb function underly partially independent neural control mechanisms.^33^ Similar results have been previously reported on a group-level by Solaro *et al*. in 11 pwMS and seven persons with clinically isolated syndrome.^21^ We were able to expand these findings by documenting that these abnormalities are present in over half of observations with normal gross dexterity and grip strength. Further, compared to Solaro et al., we found not only abnormalities in movement patterns, but also in grip force control.

### Limitations

We identified abnormalities in the digital health metrics in pwMS that have normal gross dexterity and grip strength, but did not control for other aspects of upper limb function, such as sensory impairments or fine dexterity, which might affect performance in the VPIT. Additionally, similar to most technology-based assessment paradigms, the duration and distance of the performed goal-directed movements can slightly vary within and between participants. While this might affect the estimates of the digital health metrics describing, for example, movement smoothness, this influence is expected to be minor given that the estimates of the digital health metrics are aggregated across multiple movements and task repetitions. Also, this work purposefully focused on pwMS with mild to moderate upper limb disability, which is the main target population of end-effector-based assessments such as the VPIT. Thus, upper limb movement patterns and hand grip forces in pwMS with severe upper limb disability need to be characterized in future work with adequate assessment platforms, such as robotic exoskeletons.^11^

## Conclusions

This work provides a first systematic documentation of upper limb movement patterns and hand grip forces in pwMS with mild to moderate upper limb disability. These results provide a normative range of values from digital health metrics for pwMS and thus pave the way for detecting early signs of MS using digital health metrics. Also, this work presents evidence that paves the way for a systematic application of digital health metrics in pwMS. In the future, the promising use-case of using digital health metrics to detect subtle subclinical changes in movement patterns due to rehabilitation or disease progression should be explored.

## Supplemental Material

sj-docx-1-mso-10.1177_20552173221116272 - Supplemental material for Goal-directed upper limb movement patterns and hand grip forces in multiple sclerosis Click here for additional data file. Supplemental material, sj-docx-1-mso-10.1177_20552173221116272 for Goal-directed upper limb movement patterns and hand grip forces in multiple sclerosis by Christoph M Kanzler, Ramona Sylvester, Roger Gassert, Jan Kool, Olivier Lambercy and Roman Gonzenbach in Multiple Sclerosis Journal – Experimental, Translational and Clinical
