# Supplementary material for: Goal-directed upper limb movement patterns and hand grip forces in multiple sclerosis
Source: Mult Scler J Exp Transl Clin. 2022 Aug 11;8(3):20552173221116272. doi: 10.1177/20552173221116272 (PMC9380226; doi:10.1177/20552173221116272)
Supplement: sj-docx-1-mso-10.1177_20552173221116272 - Supplemental material for Goal-directed upper limb movement patterns and hand grip forces in multiple sclerosis [file sj-docx-1-mso-10.1177_20552173221116272.docx]

**SUPPLEMENTARY MATERIALS**

**Title: Goal-directed upper limb movement patterns and hand grip forces in multiple sclerosis**

**Authors:**

Christoph M. Kanzler^1,2^, Ramona Sylvester^3^, Roger Gassert^1,2^, Jan Kool^3^, Olivier Lambercy^1,2^*, Roman Gonzenbach^3^*

*authors contributed equally.

1 Rehabilitation Engineering Laboratory, Institute of Robotics and Intelligent Systems, Department of Health Sciences and Technology, ETH Zurich, Zurich, Switzerland.

2 Future Health Technologies, Singapore-ETH Centre, Campus for Research Excellence And Technological Enterprise (CREATE), Singapore.

3 Rehabilitation Center Valens, Valens, Switzerland.

**Corresponding author:** Christoph M. Kanzler, [relab.publications@hest.ethz.ch](mailto:relab.publications@hest.ethz.ch)

Methods:

*Virtual Peg Insertion Test:* A peg can be picked up by first spatially aligning the virtual cursor, corresponding to the position and orientation of the haptic device, with the peg and afterwards applying and maintaining a hand grip force of at least 2N. After moving the peg into the hole, the peg can be released by reducing the hand grip force below 2N. A peg can be dropped on the pegboard if the force drops below the threshold during transport. Color coding is implemented to show the status of the virtual cursor, namely without lifted peg (yellow), peg lifted (green), and grip force higher than 2 N but no peg lifted (red). The haptic device provides a vertical resistance as soon as the cursor contacts the pegboard. This resistance is absent at the position of the virtual holes, thereby providing an intuitive haptic feedback that improves task perception (Fluet, Lambercy, and Gassert 2012). The VPIT is performed in a standardized seated position with approximately 90° elbow flexion, 45° shoulder abduction, and 10° shoulder flexion. Sensor data are recorded at 1 kHz.

*Virtual Peg Insertion Test metrics:* Smooth movements are a hall-mark of goal-directed movements in primate and are typically identified through a symmetric bell-shaped velocity profile (Scott 2004; Balasubramanian et al. 2015). In neurological disorders, velocity profiles with multiple submovements were observed that are expected to be indicative of abnormal motor control (Rohrer et al. 2004). In the VPIT, movement smoothness is quantified using the normalized logarithmic jerk metric (*log jerk transport/return) as well as* the spectral arc length metric (*SPARC return*), two well-established state-of-the-art approaches (Balasubramanian et al. 2015; Kanzler et al. 2020). Further, goal-directed movements of able-bodied persons are typically close to a straight line between start and target, whereas persons with neurological disorders are known to have spatial deviations from the shortest path (Cirstea and Levin 2000). In the VPIT, suboptimal movement efficiency is expressed by calculating the ratio between the shortest distance between start and target and the actually covered distance (*path length ratio transport*) (Cirstea and Levin 2000; Kanzler et al. 2020). Further, persons with neurological disorders typically have slower goal-directed movements than able-bodied controls, which is quantified through the maximum velocity metric in the VPIT (*velocity max. return*)*.* In addition to these metrics describing the movement patterns, three metrics were calculated to describe the smoothness of grip force control. For this purpose, we relied especially on the change in grip force (*force rate) to* describe dynamic adaptations during the task, which are relevant for performing daily life activities and have previously been shown to be affected in neurological disorders (Kanzler et al. 2020; Schwarz et al. 2019). More specifically, we described the number of peaks in the grip force rate profile (*grip force rate num. peaks transport*) and calculated the spectral arc length of the grip force rate during goal-directed (*grip force rate SPARC transport*) and fine movements (*grip force rate SAPRC hole approach).*

Given that the metrics typically have different units and ranges, we applied a previously introduced normalization procedure that normalizes the ranges and enables intuitive comparisons between metrics. The normalization starts by expressing each metric as a statistical distance relative to a reference population of 120 able-bodied controls between 20 and 80 years using the median absolute deviation (Kanzler et al. 2020). This was implemented using mixed effect models that cancel the effect of age, sex, and the tested body side (Kanzler et al. 2020). Afterwards, all metrics are normalized with respect to the worst performing participant that was previously available in the VPIT database (Kanzler et al. 2020). This maps all metrics on an intuitively understandable scale on the open interval ]-100%,+100%[, where 0% is the median of the reference population, 100% is the worst performing participant from the VPIT database, and negative values indicate task performance better than the median of the reference population.

*Data analysis – time series:* In order to allow a presentation of time-series on the population level, all signals were first interpolated to have equal length and then averaged within participants and subsequently across participants. This was implemented for all pwMS and compared to the signals from an age-matched able-bodied reference population. This operation was applied only for the purpose of visualizing and statistically analyzing the time-series. For all analyses referring to the digital health metrics, the time-series were not interpolated to uniform length.

*Data analysis – mixed effects models:* To analyze the influence of impaired aspects of upper limb movement patterns and hand grip forces on the VPIT task completion time, a mixed effect model was constructed, relying on the VPIT task completion time (as normalized VPIT score) as dependent variable and the digital health metrics (normalized VPIT scores) as fixed effects. In addition, potential intra-participant correlations stemming from including the left and right body side were compensated by using a participant identifier as random effect. In order to judge whether the dataset is suited for such an analysis, the typical model assumption were evaluated: The variance inflation factor (VIF) was calculated to evaluate potential co-linearities between independent variables, with a VIF of at least ten indicating strong co-linearity (Salmerón, García, and García 2018). Also, the normality of residuals and the assumption of heteroscedasticity was evaluated through visual inspection. The statistical significance of the fixed effect was then evaluated with a *F*-test.

Data analysis – *correlation analysis*: In order to evaluate whether the observed reduction in movement speed stems from weakness, a correlation analysis between the *velocity max. return* metric and grip strength as measured by the Jamar dynamometer as a surrogate marker of overall upper limb weakness was performed. The correlation analysis was implemented using Pearson correlation coefficients.

Results:

The correlation between *velocity max. return* and grip strength was -0.14 and statistically significant (p<0.05).

The VIF across metrics was 2.5±0.7 (2.0-3.6), reported as median±inter-quartile range (minimum-maximum), thereby being well below the threshold of 10 and indicating that co-linearity of independent variables was not an issue for this dataset. The visual inspection of residual and normal probability plots (Figure SM1) found no severe violation of heteroscedasticity and normality of the residuals.

**Table SM1: Detailed results of the mixed effect model analysis.** The VPIT task completion time was modeled using the VPIT metrics as fixed effects and the participant ID as random effect. The coefficients, standard (std.) errors, t- and p-values of the fixed effects are reported herein. Four metrics (log jerk TP, path length ratio TP, velocity max. RT, and force rate SPARC HA) significantly influenced the task completion time.

|  | Coefficient | Std. Error | *t*-value | *p*-value |
| --- | --- | --- | --- | --- |
| Log jerk TP | 0.11 | 0.05 | 2.07 | <0.05 |
| Log jerk RT | 0.00 | 0.04 | 0.01 | 0.99 |
| SPARC RT | -0.03 | 0.04 | -0.90 | 0.37 |
| Path length ratio TP | 0.45 | 0.07 | 6.27 | <0.001 |
| Velocity max. RT | 0.12 | 0.03 | 3.69 | <0.001 |
| Force rate num. peaks TP | 0.02 | 0.07 | 0.28 | 0.77 |
| Force rate SPARC TP | 0.04 | 0.04 | 0.89 | 0.37 |
| Force rate SPARC HA | 0.32 | 0.05 | 7.16 | <0.001 |

*
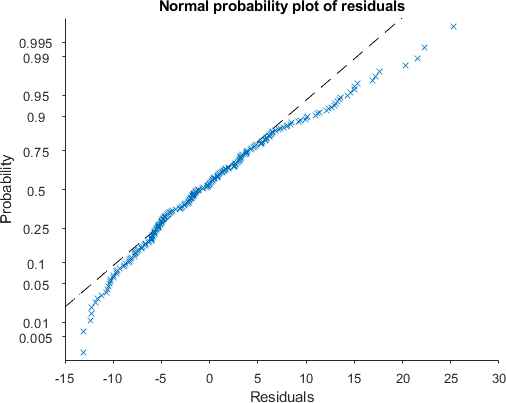

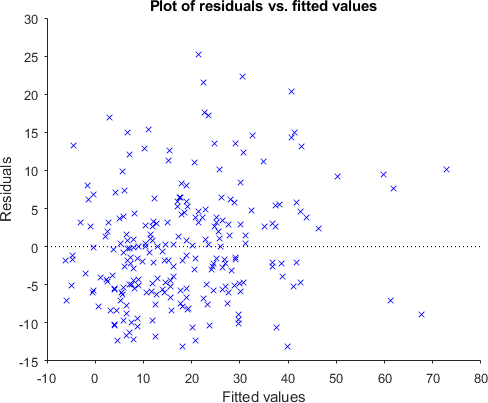
*

A

B

**Figure SM1: Model diagnostics for the linear regression analysis.** Residuals were visualized against the fitted values to evaluate heteroscedasticity (A) and normal probability plots to evaluate the normality of the residuals (B).

**References**

Balasubramanian, Sivakumar, Alejandro Melendez-Calderon, Agnes Roby-Brami, and Etienne Burdet. 2015. “On the Analysis of Movement Smoothness.” *Journal of Neuroengineering and Rehabilitation* 12 (1): 112. https://doi.org/10.1186/s12984-015-0090-9.

Cirstea, M. C., and M F Levin. 2000. “Compensatory Strategies for Reaching in Stroke.” *Brain : A Journal of Neurology* 123 (5): 940–53. https://doi.org/10.1093/brain/123.5.940.

Fluet, Marie Christine, Olivier Lambercy, and Roger Gassert. 2012. “Effects of 2D/3D Visual Feedback and Visuomotor Collocation on Motor Performance in a Virtual Peg Insertion Test.” *Proceedings of the Annual International Conference of the IEEE Engineering in Medicine and Biology Society, EMBS*, 4776–79. https://doi.org/10.1109/EMBC.2012.6347035.

Kanzler, Christoph M, Mike D Rinderknecht, Anne Schwarz, Ilse Lamers, Cynthia Gagnon, Jeremia P. O. Held, Peter Feys, Andreas R Luft, Roger Gassert, and Olivier Lambercy. 2020. “A Data-Driven Framework for Selecting and Validating Digital Health Metrics: Use-Case in Neurological Sensorimotor Impairments.” *Npj Digital Medicine* 3 (1): 80. https://doi.org/10.1038/s41746-020-0286-7.

Rohrer, Brandon, Susan Fasoli, Hermano Igo Krebs, Bruce Volpe, Walter R. Frontera, Joel Stein, and Neville Hogan. 2004. “Submovements Grow Larger, Fewer, and More Blended during Stroke Recovery.” *Motor Control* 8 (4): 472–83. https://doi.org/10.1123/mcj.8.4.472.

Salmerón, R., C. B. García, and J. García. 2018. “Variance Inflation Factor and Condition Number in Multiple Linear Regression.” *Journal of Statistical Computation and Simulation* 88 (12): 2365–84. https://doi.org/10.1080/00949655.2018.1463376.

Schwarz, Anne, Christoph M. Kanzler, Olivier Lambercy, Andreas R. Luft, and Janne M. Veerbeek. 2019. “Systematic Review on Kinematic Assessments of Upper Limb Movements After Stroke.” *Stroke* 50 (3): 718–27. https://doi.org/10.1161/STROKEAHA.118.023531.

Scott, Stephen H. 2004. “Optimal Feedback Control and the Neural Basis of Volitional Motor Control.” *Nature Reviews Neuroscience* 5 (7): 532–46. https://doi.org/10.1038/nrn1427.
